# Address in Surgery

**Published:** 1903-08-08

**Authors:** 


### ADDRESS IN SURGERY.

Mr. Mayo Robson, who delivered the address in surgery, devoted himself to observations on the evolution of abdominal surgery from personal reminiscences extending over a third of a century and the performance of 2,000 operations. Commencing with a historical survey of the operation of ovariotomy from its first performance up to the present time, when the mortality of the operation in general hospitals is only about 4·4 per cent., he passed on to the consideration of other branches of abdominal surgery. The two great steps of advance which have been made in all abdominal procedures have been, firstly, the innovation of antiseptic surgery; and secondly, Lawson Tait's demonstration of the value of natural drainage through the intestines, and the importance of the avoidance of morphine. Lawson Tait made a routine practice of purging after operation, and so avoided that distension which used to kill so rapidly in early ovarian work. Had purging and the avoidance of morphine been adopted in the early seventies, Mr. Mayo Robson believed that the large mortality of that day would have been materially lessened. Over and over again as a student he had seen patients killed by intestinal paralysis induced through morphine, who in the light of recent knowledge might have been saved by a dose of calomel and Epsom salts.

What gives special interest to surgical affections of the abdomen is the fact that the viscera are invested in whole or in part by the large serous peritoneal sac, and that infection, however communicated, is apt to assume serious proportions, involving not only the whole abdominal cavity, but the system at large. In the seventies, and even much later, the diagnosis of peritonitis used to be made with great satisfaction, as if it were final and sufficient; but who at the present time with any claim to scientific knowledge would be content with a mere name that gives no idea of the disease to be treated or of the cause of the illness?

The discovery of the frequency of perforative appendicitis, perforative gastric ulcer, and of other peritoneal catastrophes is gradually abolishing the term "acute peritonitis" for the more rational one of "acute peritoneal infection," for the treatment of which surgery alone is of any use, and even for that to be of service operation must not be long delayed. Could anything show this more definitely than the results of treatment of perforated gastric ulcers, which, if operated on at once, would have hardly any mortality, if within 12 hours of rupture would have a mortality of 16·6 per cent., if within 24 hours 63 6 per cent., if within 36 hours 87·5 per cent., while operation delayed for 48 hours will only rarely succeed—or, to give another instance, than the results of surgical treatment of the intestine in typhoid fever, alas! too seldom resorted to? To give an example: Out of a total number of 900 cases of typhoid fever occurring in St. Bartholomew's Hospital in the years 1895 to 1901 there were 102 deaths. Among the 900 cases were 38 of perforation, of which 34 died unoperated upon, whereas of the four operated on three recovered. As spontaneous recovery from perforation is extremely rare, it is of the utmost importance that not only should we have an early recognition of the perforation, but also an immediate operation. The same is true of other perforative lesions such as occur in the appendix and gall-bladder; and here also, though much advance has been made, there is still further room for improvement, mainly in the direction of earlier operation.

Hysterectomy for myoma had been the subject of much controversy and acrimonious debate; for while, on the one hand, it has been urged that uterine myoma is not a fatal disease and rarely requires surgical treatment, on the other it has been shown that the disease is not only often fatal, but that in many cases medical treatment is absolutely futile, and that many of the victims of uterine fibroids, unless treated radically, are condemned to life-long invalidism, often ending in death. As Dr. Cullingworth, Mrs. Scharlieb, and others have shown so convincingly, it is quite a mistake to say that the symptoms usually cease with the menopause. The contrast presented by those patients who have been operated upon, and who are, as a rule, completely restored to health in a few weeks, with the chronic invalids who show their disease in their bloodless features, and have to spend half their time in bed, is very marked; and Mr. Robson is convinced that where the symptoms of hæmorrhage and pain and other distressing complications supervene, the old views ought to be discarded and operation advised, for there is nothing in the whole range of medicine to be compared with the disappointment of a woman who, having spent 20 to 30 years of misery as a chronic invalid under the idea that her troubles will cease at the menopause, finds that her dream of health has been a "will o' the wisp," and that her life can only be saved even then by the operation she has been living to avoid. As to the best form of operation in these cases, it is better to treat each case on its merits.

Other branches of surgery which are full of interest, and in which great progress has been made in recent years, are those comprising the surgery of the bile-passages and liver, of the stomach, pancreas, and intestines, of the kidneys, ureters, and bladder.

Although abdominal surgery in its widest sense has practically sprung into being and developed within only the last third of a century, something more than merely crossing the threshold has been accomplished, though very much still remains to be done; the diligent student has no need to lament with Alexander and bemoan that there are no more worlds to conquer.

